# Whole-Genome Sequencing for Resistance Level Prediction in Multidrug-Resistant Tuberculosis

**DOI:** 10.1128/spectrum.02714-21

**Published:** 2022-06-06

**Authors:** Jinli Li, Tingting Yang, Chuangyue Hong, Zheng Yang, Likai Wu, Qian Gao, Hui Yang, Weiguo Tan

**Affiliations:** a Shenzhen Center for Chronic Disease Control, Shenzhen, China; b Key Laboratory of Medical Molecular Virology (MOE/NHC/CAMS), School of Basic Medical Sciences, Shanghai Medical College, Shanghai Institute of Infectious Disease and Biosecurity and Shanghai Public Health Clinical Center, Fudan University, Shanghai, China; Instituto Oswaldo Cruz

**Keywords:** minimal inhibitory concentration (MIC), multidrug-resistant tuberculosis (MDR-TB), resistance level, whole-genome sequencing (WGS)

## Abstract

Defining the precise relationship between resistance mutations and quantitative phenotypic drug susceptibility testing will increase the value of whole-genome sequencing (WGS) for predicting tuberculosis drug resistance. However, a large number of WGS data sets currently lack corresponding quantitative phenotypic data—the MICs. Using MYCOTBI plates, we determined the MICs to nine antituberculosis drugs for 154 clinical multidrug-resistant tuberculosis isolates from the Shenzhen Center for Chronic Disease Control in Shenzhen, China. Comparing MICs with predicted drug-resistance profiles inferred by WGS showed that WGS could predict the levels of resistance to isoniazid, rifampicin, streptomycin, fluoroquinolones, and aminoglycosides. We also found some mutations that may not be associated with drug resistance, such as EmbB D328G, mutations in the *gid* gene, and C−12T in the *eis* promoter. However, some strains carrying the same mutations showed different levels of resistance to the corresponding drugs. The MICs of different strains with the RpsL K88R, *fabG1* C−15T mutations and some with mutations in *embB* and *rpoB*, had MICs to the corresponding drugs that varied by 8-fold or more. This variation is unexplained but could be influenced by the bacterial genetic background. Additionally, we found that 32.3% of rifampicin-resistant isolates were rifabutin-susceptible, particularly those with *rpoB* mutations H445D, H445L, H445S, D435V, D435F, L452P, S441Q, and S441V. Studying the influence of bacterial genetic background on the MIC and the relationship between rifampicin-resistant mutations and rifabutin resistance levels should improve the ability of WGS to guide the selection of medical treatment regimens.

**IMPORTANCE** Whole-genome sequencing (WGS) has excellent potential in drug-resistance prediction. The MICs are essential indications of adding a particular antituberculosis drug dosage or changing the entire treatment regimen. However, the relationship between many known drug-resistant mutations and MICs is unclear, especially for rarer ones. The results showed that WGS could predict resistance levels to isoniazid, rifampicin, streptomycin, fluoroquinolones, and aminoglycosides. However, some mutations may not be associated with drug resistance, and some others may confer various MICs to strains carrying them. Also, 32.3% of rifampicin (RIF)-resistant strains were classified as sensitive to rifabutin (RFB), and some mutations in the *rpoB* gene may be associated with this phenotype. Our data on the MIC distribution of strains with some rarer mutations add to the accumulated data on the resistance level associated with such mutations to help guide further research and draw meaningful conclusions.

## INTRODUCTION

Multidrug-resistant tuberculosis (MDR-TB) severely hampers global tuberculosis prevention and control. In 2019, there were approximately 550,000 new rifampicin-resistant (RR) tuberculosis cases, of which 78% were also resistant to isoniazid (INH) and therefore MDR-TB (1). Compared with the treatment for drug-sensitive tuberculosis, the treatment for MDR-TB is longer and more expensive, and the percentage of patients who are ultimately cured is lower. Inadequate or inappropriate treatment of MDR-TB risks the acquisition of resistance to additional drugs and prolongs the time during which the patient can transmit the resistant strain within their community (2). To maximize the effectiveness of treatment and minimize the development of additional resistance, accurate drug susceptibility testing (DST) should be performed on all Mycobacterium tuberculosis clinical isolates (3). Whole-genome sequencing (WGS) has the potential to detect drug resistance more rapidly than traditional phenotypic DST without the need for biological safety infrastructure and can accurately predict resistance to the full range of antituberculosis drugs (4, 5). Some countries, such as the United Kingdom and the Netherlands, have implemented WGS-guided individualized treatment (6) and WGS-based monitoring of all tuberculosis patients (7).

Although several studies have shown that different drug-resistance mutations are associated with different levels of drug resistance (8–10), the association is not perfect. Strains with identical resistance mutations can have different MICs, in some cases ranging from sensitive to highly resistant. MICs are important indications of the need to increase the dosage of a particular antituberculosis drug or alter the entire treatment regimen, and therefore WGS would be more valuable if there were more information on the relationship between resistance-associated mutations and MICs (8, 9). To provide more information, we selected 154 MDR-TB strains from the Shenzhen Center for Chronic Disease Control (CCDC) tuberculosis laboratory and compared their MICs determined by phenotypic DST with the resistance-associated mutations found by WGS.

## RESULTS

### Strains included in analysis.

From the tuberculosis reference laboratory of Shenzhen CCDC, we selected 182 M. tuberculosis strains isolated from patients during 2013 through 2019 and determined to be MDR-TB—meaning they are resistant to at least isoniazid and rifampicin—using the proportion method or molecular tests. All 182 isolates were subjected to whole-genome sequencing and phenotypic MIC determination. Fifteen isolates were presumed to be duplicates because they were very similar to other strains and had the same resistance-associated mutations and were therefore excluded. Among the remaining 167 strains, 13 strains were excluded because the results of the MIC determination with MYCOTBI plates did not match the prior results using the proportion method (Table S1). Of the remaining 154 MDR-TB isolates that were included in this study, 136 (88.3%) belonged to lineage 2, 17 (11.0%) to lineage 4, and 1 strain to lineage 1 (Fig. S1). The genomes of the 154 MDR-TB strains were sequenced with a median depth of 198× (interquartile range, 89 to 395×) and a median coverage of 99.2% (interquartile range, 99.1 to 99.3%).

### Drug susceptibility results based on MIC versus WGS.

**FIG 1:**
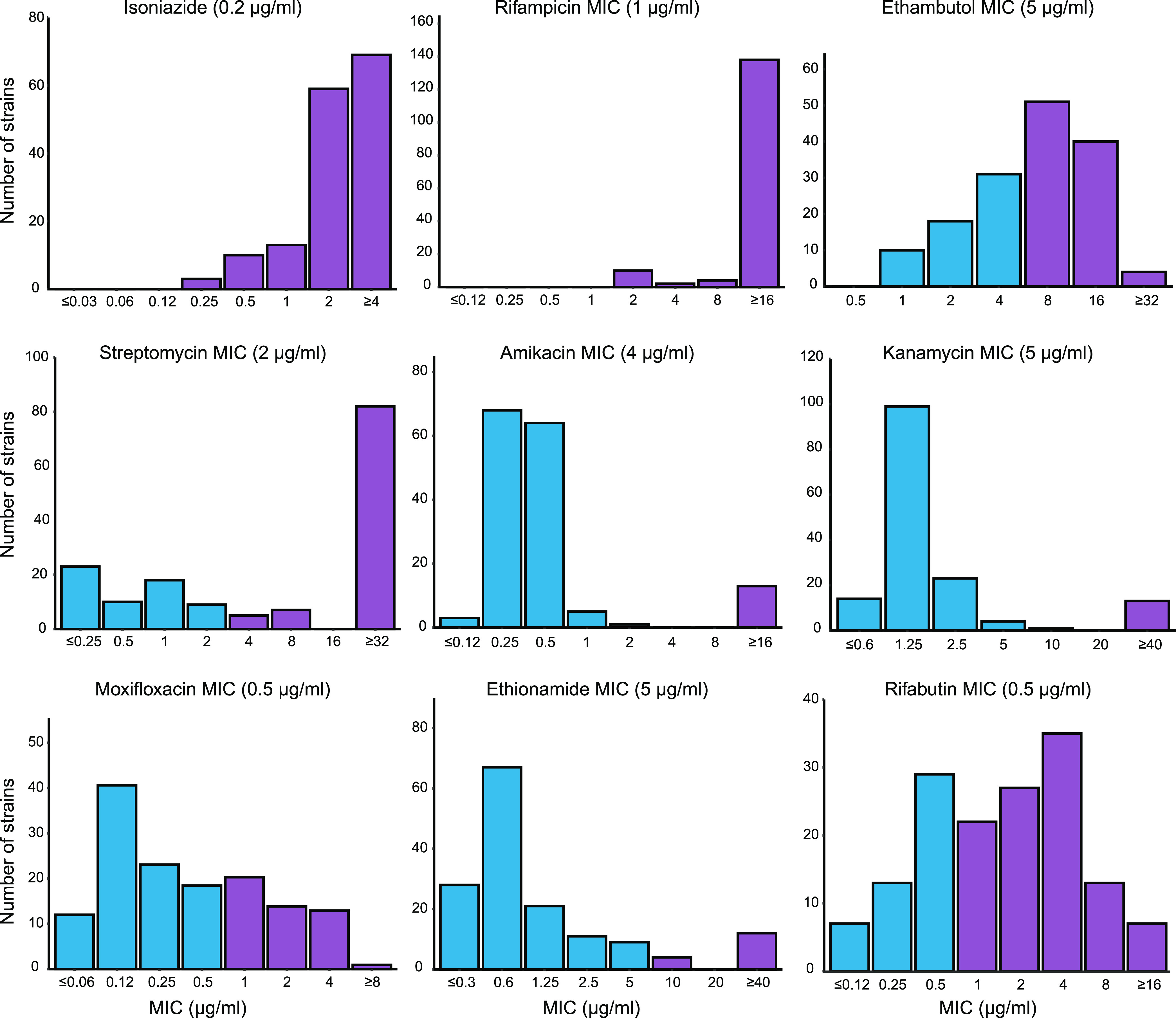
The MIC distribution of the 154 multidrug-resistant tuberculosis strains against nine antituberculosis drugs. Purple indicates drug-resistant strains with MICs greater than the breakpoint, which is specified in parentheses above each panel.

MYCOTBI plates were used to determine the MICs of the MDR-TB strains against 12 antituberculosis drugs (Table S2). *para*-Aminosalicylic acid (PAS) and cycloserine (CS) were excluded from the analysis because the phenotypic DST results for these drugs are unreliable (11, 12). Ofloxacin (OFX) was excluded because it is no longer used for tuberculosis treatment, leaving moxifloxacin (MOX) as the only fluoroquinolone. The MICs of the 154 MDR strains for the remaining 9 drugs are shown in Fig. 1. There were 95 (61.7%), 52 (33.8%), 94 (61.0%), 14 (9.1%), 13 (8.4%), 16 (10.4%), and 105 (68.2%) strains resistant to ethambutol (EMB), moxifloxacin (MOX), streptomycin (SM), kanamycin (KM), amikacin (AMK), ethionamide (ETH), and rifabutin (RFB), respectively (Table S3). The prediction of drug resistance by WGS largely agreed with the phenotypic drug sensitivity based on the MIC (Table 1). Compared to the MIC-based results, the sensitivity of WGS for detecting resistance to each of the nine drugs was 93% or above. With the exception of EMB, SM, and RFB, the specificities of WGS prediction for the other drugs were above 70%. Overall, in determining whether a strain was sensitive or resistant, the two methods agreed in more than 80% of strains for all drugs except RFB.

**TABLE 1:** The accuracy of whole-genome sequencing in predicting drug resistance, compared to MIC determinations^*a*^

Drug	Resistant phenotype			Susceptible phenotype			Sensitivity	Specificity	PPV	NPV	Agreement
	R	ND	Total	R	ND	Total					
Isoniazid	152	2	154	0	0	0	98.7		100.0	0.0	98.7
Rifampicin	153	1	154	0	0	0	99.4		100.0	0.0	99.4
Ethambutol	91	4	95	25	34	59	95.8	57.6	78.4	89.5	81.2
Streptomycin	93	0	93	21	40	61	100.0	65.6	81.6	100.0	86.4
Ethionamide^*b*^	15	1	16	22	114	136	93.8	83.8	40.5	99.1	84.7
Amikacin	13	0	13	0	141	141	100.0	100.0	100.0	100.0	100.0
Rifabutin^*c*^	103	1	104	49	0	49	99.0	0.0	67.8	0.0	67.3
Kanamycin	14	0	14	6	134	140	100.0	95.7	70.0	100.0	96.1
Moxifloxacin	50	2	52	26	76	102	96.2	74.5	65.8	97.4	81.8

*^a^*If drug-resistance mutation is detected in a sample, the sample is designated resistant (R) to the drug; otherwise, the prediction result is “no resistance mutations detected” (ND). NPV, negative predictive value; PPV, positive predictive value.

*^b^*Default for two strains.

*^c^*Default for one strain.

### Relationship between different resistance-associated mutations and MICs.

To investigate the relationship between resistance-associated mutations and resistance levels to the nine drugs, we compared the specific mutations with the MICs of the 154 MDR-TB isolates and found that different drug-resistance mutations were associated with different MIC ranges (Table 2; Table S4). For INH, nonsynonymous mutations in the *katG* gene generally resulted in high-level resistance (MIC ≥ 1 μg/mL), but a strain carrying the KatG Q127P mutation showed low-level resistance (Fig. S2). Mutations in the *inhA* and *ahpC* promoters were also associated with low levels of INH resistance, but when they occurred together with *katG* gene mutations, the strains had high-level INH resistance. The MICs of strains carrying both KatG S315T and *inhA* promoter mutations were at least 4 μg/mL (Table 2; Fig. S2).

**TABLE 2:** Drug-resistance mutations with corresponding MICs for nine anti-tuberculous drugs^*a*^

Drug (breakpoint)	Gene	Mutation	MIC (μg/mL)							
Isoniazide (0.2 μg/mL)			**0.03**	**0.06**	**0.12**	**0.25**	**0.5**	**1**	**2**	**4**
	*katG*	S315T/S315N				**1**	**3**	**11**	**54**	**51**
		Other				**1**		**1**		**2**
	*inhA*	C−15T				**1**	**4**		**3**	
		Other					**1**		**1**	
	*ahpC*	*ahpC* promoter mutation					**1**			**1**
	*katG* and *inhA*	*katG* 315 and *inhA* promoter mutation								**11**
		Other					**1**		**1**	**2**
	*katG* and *ahpC*	*katG* frameshift and *ahpC* C−72T								**1**
	ND							**1**		**1**
Rifampicin (1 μg/mL)			**0.12**	**0.25**	**0.5**	**1**	**2**	**4**	**8**	**16**
	*rpoB*	S450L/S450W/S450F								**94**
		H445Y/H445D/H445L/H445N/H445R/H445S					**3**	**1**	**1**	**13**
		D435V/D435F					**1**		**1**	**4**
		Other single mutation					**3**	**1**		**1**
		S450L and other							**1**	**10**
		L430P and other					**2**			**10**
		Other multiple mutations					**1**		**1**	**5**
	ND									**1**
Ethambutol (5 μg/mL)			**0.5**	**1**	**2**	**4**	**8**	**16**	**32**	
	*embB*	M306V/M306I/M306L				12	**27**	**25**	**3**	
		G406A/G406S/G406D		1	1	4	**9**	**2**		
		Q497R/Q497K				1	**6**	**3**		
		Other single mutation			1	1	**1**	**1**		
		M306I and G406D/D354A/G406A/Q497R				3	**1**			
	*embA*	Promoter mutation				1	**2**			
	*embA* and *embB*	*embA* promoter and *embB* mutation					**3**	**7**	**1**	
	ND			9	16	9	**2**	**2**		
Moxifloxacin (0.5 μg/mL)			**0.06**	**0.12**	**0.25**	**0.5**	**1**	**2**	**4**	**8**
	*gyrA*	D94G/D94A/D94N/D94H/D94Y		1	1	5	**10**	**14**	**9**	**1**
		A90V		1	4	12	**8**			
		S91P					**3**	**1**		
		A90V and D94G							**1**	
	*gyrB*	A504V			1					
	*gyrA* and *gyrB*	*gyrA* 94 and *gyrB* mutation		1					**2**	
		*gyrA* H70R and *gyrB* E501D							**1**	
	ND		13	41	19	3	**1**		**1**	
Streptomycin (2 μg/mL)			**0.25**	**0.5**	**1**	**2**	**4**	**8**	**16**	**32**
	*rpsL*	K43R								**73**
		K88R	1	1	3		**3**	**5**		**4**
	*rrs*	A514C			4	4	**1**	**1**		
		Other			1	1		**1**		
	*gid*	*gid* mutation			4					
	*rpsL* and *rrs*	*rpsL* and *rrs* mutation				1				**4**
	*rpsL* and *gid*	*rpsL* K43R and *gid* 115_del_G								**1**
	ND		23	8	6	3	**1**			
Amikacin (4 μg/mL)			**0.12**	**0.25**	**0.5**	**1**	**2**	**4**	**8**	**16**
	*rrs*	A1401G								**13**
	ND		3	68	64	5	1			
Kanamycin (5 μg/mL)			**0.6**	**1.2**	**2.5**	**5**	**10**	**20**	**40**	
	*rrs*	A1401G							**13**	
	*eis*	Promoter mutation		1	1	4	**1**			
	ND		14	98	22					
Ethionamide (5 μg/mL)			**0.3**	**0.6**	**1.25**	**2.5**	**5**	**10**	**20**	**40**
	*ethA*	Frameshift		1	6	2		**1**		**1**
		Other		1		1				
	*inhA*	I21T								**1**
	*fabG1*	C−15T			1	3	5	**1**		**5**
		T−8C		2						
	*fabG1* and *ethA*	*fabG1* C−15T and *ethA* frameshift								**3**
	*fabG1* and *InhA*	*fabG1* C−15T and *InhA* I21T						**1**		**2**
	ND		28	63	14	5	4	**1**		
Rifabutin (0.5 μg/mL)			**0.12**	**0.25**	**0.5**	**1**	**2**	**4**	**8**	**16**
	*rpoB*	S450L/S450W/S450F		2	18	**13**	**24**	**23**	**9**	**5**
		H445Y/H445D/H445L/H445N/H445R/H445S	1	4	4	**2**		**3**	**1**	**2**
		D435F/D435V	2	1	2		**1**			
		Other single mutation	2	1	1	**1**				
		S450L and other		3		**2**	**1**	**4**	**1**	
		L430P and other	1	1	1	**3**	**1**	**4**	**1**	
		Other multiple mutations	1	1	3			**1**	**1**	
	ND					**1**				

*^a^*The numbers indicate the number of strains with this MIC. The numbers in bold type indicate resistance MICs above the breakpoint, shown in parentheses. ND, no resistance mutations detected.

For rifampicin (RIF), strains with the common *rpoB* mutations in codons 450, 445, and 435 and *rpoB* double mutations generally showed high-level resistance (MIC ≥ 8 μg/mL), while strains harboring the H445L (2 strains), H445S (1 strain), and D435F (1 strain) mutations were associated with low-level resistance (≤4 μg/mL) (Table 2; Fig. S3). All 12 strains with the RpoB L430P mutation also carried other RpoB mutations, most commonly in codons 435 (6 strains) and 445 (3 strains). The double mutant strains were highly resistant to RIF, except for strains with the L430P + M434I or L430P + S493L mutations (Fig. S3).

EMB resistance mutations mainly occurred in *embB* and were associated with varied MICs (Table 2; Fig. S4). For example, the MICs of strains carrying the M306V, M306I, M306L, M406A, and M406S mutations differed by 4- to 8-fold. Of the strains with these mutations, 3 of 42 (7%) with M306V, 7 of 19 (36.8%) with M306I, 2 of 6 (33.3%) with M306L, 2 of 9 (22.2%) with M406A, and 1 of 4 (25%) with M406S mutations had MICs below the breakpoint for EMB (Fig. S4). This demonstrates that even strains with these high-confidence EMB resistance-associated mutations can be phenotypically sensitive, perhaps because the breakpoint (5 μg/mL) for EMB falls between two concentrations (4 and 8 μg/mL) tested on the MYCOTBI plates. Of 10 strains with EmbB G406A mutation, 7 had MICs of 8 μg/mL, indicating low-level EMB resistance, but strains with the EmbB G406D (3) and D328G (2) mutations were sensitive to EMB, suggesting that these may not be associated with EMB resistance.

Most strains that were resistant to the fluoroquinolones had a mutation in either the *gyrA* or *gyrB* genes, most frequently altering *gyrA* codons 90 or 94 (Table S4). Strains with the GyrA D94G, D94H, D94N, and D94Y mutations showed high-level MOX resistance (MIC ≥ 2 μg/mL), while strains with the D94A and A90V mutations had low-level resistance (Table 2; Fig. S5). The MICs of strains with the GyrA D94A or A90V mutations include the breakpoint of MOX, consistent with the World Health Organization (WHO)’s classification of the two mutations as conferring low-level resistance (13).

SM resistance mutations occurred mainly in *rpsL* (Table S4). Strains with the RpsL K43R mutation showed high-level SM resistance (MIC ≥ 32 μg/mL), while the MICs of strains with the K88R mutation varied from ≤0.25 to ≥32 μg/mL (Table 2; Fig. S6). Of the 10 strains with the *rrs* A514C mutation, 8 have MICs equal to (4 strains) or below (4 strains) the breakpoint. Because the *rrs* A514C is regarded as a high-confidence SM resistance-associated mutation, it is possible that there was a problem with the MIC determination, or the level of resistance conferred by this mutation, for unknown reasons, varies in different strains. The MICs of all 4 strains with *gid* mutations were below the breakpoint for SM (Table 2; Fig. S6).

The principal mutation found in strains with AMK and KM resistance was *rrs* A1401G, which caused high-level resistance to both drugs (MICs ≥ 4 or 8 times the breakpoint) (Table 2). Mutations in the promoter of the *eis* gene (7 strains) were associated with a low level of KM resistance, and the two strains with the *eis* C−12T mutation had MICs in the sensitive range (Fig. S7), suggesting it may not always be associated with KM resistance. The C−15T mutation in the *fabG1* promoter region was present in most strains with ETH resistance, but their MICs ranged from 1.25 to ≥40 μg/mL, making it difficult to define the relationship between this mutation and ETH resistance. Of 11 strains with *ethA* frameshift mutations, 9 had MICs lower than the breakpoint, so the relationship of these mutations to clinical ETH resistance is also uncertain (Table 2; Fig. S8).

### Relationship of RIF resistance mutations to RFB resistance.

Since cross-resistance to RIF and RFB is common, we further analyzed the effect of known RIF-resistant mutations on the MICs for RFB. RIF resistance mutations were detected in 152 of the 153 strains with RFB MIC results, but the effect was often different for the two antibiotics. The RpoB resistance mutations generally increased the MICs for RIF more than for RFB (Fig. S9). In strains with the RpoB S450L mutation, which confers high-level RIF resistance, the RFB MICs ranged from 0.25 to ≥16 μg/mL, and the 2 strains with the RpoB S450L mutation and RFB MICs of 0.25 μg/mL were classified as RFB-sensitive (Table 3). Overall, of the 152 RIF-resistant strains, there were 49 (32.3%) that were classified as RIF-resistant/RFB-sensitive (Fig. S10), particularly those with the RpoB mutations H445D, H445L, H445S, D435V, D435F, L452P, S441Q, and S441V (Table 3; Fig. S3 and S11).

**TABLE 3:** RpoB mutations and MICs to rifampicin and rifabutin in 49 rifampicin-resistant/rifabutin-susceptible isolates

Mutation	Rifampicin (1 μg/mL breakpoint)	Rifabutin (0.5 μg/mL breakpoint)	No. of strains
RpoB_S450L	≥16	0.25	2
	≥16	0.5	18
RpoB_H445D	≥16	0.25	1
	≥16	0.5	1
RpoB_H445L	2	0.25	1
	4	0.5	1
RpoB_H445N	8	≤0.12	1
RpoB_H445R	≥16	0.5	1
RpoB_H445S	2	0.25	1
RpoB_H445Y	2	0.25	1
	≥16	0.5	1
RpoB_D435F	2	≤0.12	1
RpoB_D435V	8	0.25	1
	≥16	0.5	2
	≥16	≤0.12	1
RpoB_L452P	2	0.25	1
	2	0.5	1
RpoB_S441Q	4	≤0.12	1
RpoB_S441V	2	≤0.12	1
RpoB_L452P and RpoB_T676P	2	0.25	1
RpoB_R167C and RpoB_D435Y	8	≤0.12	1
RpoB_D435A and RpoB_L452P	≥16	0.5	1
RpoB_H445N and RpoB_L452P	≥16	0.5	2
RpoB_T399I and RpoB_S450L	8	0.25	1
RpoB_V305L and RpoB_S450L	≥16	0.25	1
RpoB_Y308D and poB_S450L	≥16	0.25	1
RpoB_L430P and RpoB_D435G	≥16	0.5	1
	≥16	≤0.12	1
RpoB_L430P and rpoB_M434I	2	0.25	1

## DISCUSSION

This study compared the MICs for 9 antituberculosis drugs with known drug-resistance mutations in 154 MDR-TB strains and found that WGS has the potential to infer resistance levels for key antituberculosis drugs, including isoniazid, rifampicin, streptomycin, fluoroquinolones, and the aminoglycosides. The common drug-resistance mutations, such as nonsynonymous *katG* mutations, RpoB S450X, H445R, H445Y, and D435V, GyrA D94H/N/G, RpoL K43R, and *rrs* A1401G were usually associated with high resistance to the corresponding drugs. Low-level resistance was generally found in strains with *fabG1* and *ahpC* promoter mutations, RpoB H445L, and also GyrA D94A and A90V. These results are consistent with the descriptions in the WHO technical manual (13).

A wealth of genomic data on drug-resistant M. tuberculosis has become available in recent years, but quantitative phenotypic DST—the actual MICs—are lacking for most of the genetic data sets. In the genomic studies that include quantitative DSTs, the relationships between some resistance-associated mutations and the MICs have been inconsistent. For example, 7 of 19 (36.8%) of our strains with the EmbB M306I and all 3 strains with the EmbB G406D mutation had MICs lower than the EMB breakpoint and were classified as sensitive. In the study of Ruesen et al. (8), the MICs of 14 strains with the EmbB M306I mutation and five with G406D mutation were all lower than the breakpoint for EMB, while Gygli et al. (9) found that strains with the same EmbB mutations all had MICs that were higher than the breakpoint. Another example is that in our study, the RpoB H445N was associated with high-level RIF resistance, but the same mutation was previously reported in strains classified as RIF-sensitive (8, 14, 15). Different methodologies and RIF MIC variations of strains carrying the mutation may account for this inconsistency (8, 13–15). This suggest that more data are needed to improve the accuracy of WGS predictions of drug-resistance levels.

Although cross-resistance between RIF and RFB is common, RIF-resistant/RFB-susceptible isolates have been previously reported (16–18). In our study, 32.3% of RIF-resistant isolates were RFB-susceptible, which is slightly higher than the proportions reported elsewhere (13 to 28%) (16–18). The mutations we found in strains that were RIF-resistant/RFB-susceptible were as previously reported D435V, D435F, H445D, H445L, and L452P (16–18). However, we also found the RIF-resistant/RFB-susceptible isolates carrying the S441Q, S441V, and H445S mutations, which have not been previously reported with this phenotype. The RpoB S441L mutation has also been associated with the RIF-resistant/RFB-susceptible phenotype (14, 15). The relationship between RIF-resistant mutations and RFB resistance levels should be studied further, as RFB might be effective in some tuberculosis patients with isolates that are resistant to RIF.

The purpose of studying the relationship between resistance-associated mutations and MICs is to improve the ability of WGS to predict resistance level and thereby guide the selection of treatment regimens without the need for phenotypic DST. However, the precision of WGS predictions is not completely reliable because some strains with the same resistance-associated mutations have different levels of resistance, ranging from high-level resistance to low-level resistance and even MICs classified as drug-sensitive (9). Different strains with the RpsL K88R and *fabG1* C−15T mutations, as well as some mutations in EmbB and RpoB, had MICs to SM, ETH, EMB, and RFB, respectively, that varied by 8-fold or more. In a phenomenon known as epistasis, the genetic background of a strain can influence the occurrence and effect of drug-resistance mutations. For example, the strain background was found to influence the evolution of fluoroquinolone resistance mutations, as well as the level of resistance the mutations conferred (19). It has also been proposed that epistatic interactions with polymorphisms in the *embABC* operon may affect the MICs of strains carrying EmbB M306I mutations (9), but the exact nature of these epistatic interactions is unknown. The influence of the bacterial genetic background on MIC levels clearly needs to be studied further. Clofazamine, linezolid, bedaquiline, and delamanid are new and repurposed drugs that play important roles in newer treatment regimens for drug-resistant tuberculosis, but we could not analyze the association of their resistance mutations with MICs because the MYCOTBI plate does not include these drugs.

In conclusion, this comprehensive analysis of 154 clinical MDR-TB strains analyzed the relationship between resistance-associated mutations and resistance levels to 9 drugs. It provides additional data on the accuracy of WGS for predicting high and low levels of resistance to INH, RIF, SM, fluoroquinolones, and aminoglycosides and confirms previously the described heterogeneity of MICs conferred by mutations associated with resistance to ethambutol.

## MATERIALS AND METHODS

### M. tuberculosis clinical isolates.

The M. tuberculosis clinical isolates were selected from the central Shenzhen City CCDC tuberculosis laboratory, which stores clinical strains collected from all 10 districts of Shenzhen. To identify MDR-TB isolates, the laboratory performs phenotypic DST for rifampin and isoniazid using the proportion method and/or molecular assays. Among the MDR-TB strains isolated during 2013 through 2019, 182 were selected for the study.

### Whole-genome sequencing.

The MDR-TB strains were recultured and sequenced as previously described (20). The raw data were uploaded to the *Mycobacterium tuberculosis* WGS data analysis platform (SAM-TB) (21) for quality control, detection of known resistance-associated mutations, lineage identification, pairwise single-nucleotide polymorphism (SNP) distance calculation, and construction of phylogenetic trees. For molecular DST, SAM-TB integrates drug-resistance mutation sets from the CRyPTIC Consortium/100,000 Genomes Project and the TB-Profiler tool (22, 23). Variant calls were based on a minimum read depth of 5× with at least one read from each strand. Drug-resistance mutations were noted when they were present on at least 10% of reads. The pairwise distance and phylogenetic analysis were analyzed using fixed SNPs (frequency ≥ 75%). Clustered strains were defined as strains that differed by 12 or fewer SNPs (24).

### Strain culture and MIC detection.

To prepare the inoculum, the cultured strains were put into special ultrasonic dispersion tubes (TB health care, Guangdong, China) with 2.5 mL sterile water for ultrasonic turbidity, and the turbidity for each strain was adjusted to 1.0 McFarland by adding more sterile water or bacterial culture as appropriate. Then, 100 μL of the liquid was removed and added to 10 mL 7H9 medium containing 10% oleic acid-albumin-dextrose-catalase (OADC). After 30 s of vortex mixing, 100 μL of the diluted bacterial suspension was pipetted into each well of the Sensititre MYCOTBI plates (Thermo Fisher, Scientific Inc., USA). The plates were incubated in an aerobic environment at 37°C for 10 days, with growth monitored for 7 to 10 days. If the strain did not grow well after 10 days, the plate was incubated for another 11 days. The VIZION system was used to read the MICs.

The MICs were obtained using 2-fold dilutions with the following concentration ranges: INH, 0.03 to 4 μg/mL; RIF, 0.12 to 16 μg/mL; EMB, 0.5 to 32 μg/mL; OFX, 0.25 to 32 μg/mL; MOX, 0.06 to 8 μg/mL; SM, 0.25 to 128 μg/mL; KM, 0.6 to 40 μg/mL; AMK, 0.12~16 μg/mL; RFB, 0.12 to 16 μg/mL; ETH, 0.3 to 40 μg/mL; PAS, 0.5 to 64 μg/mL; and CS, 2 to 256 μg/mL (Table S2). The MICs were defined as the minimal antibiotic concentrations that significantly inhibited the growth of the MDR strains compared to growth of the same strains in the positive control wells without antituberculosis drugs. H37Rv (ATCC 27294) was used as the drug-sensitive strain control on each batch tested.

The breakpoint for each drug was defined according to the CLSI M24-A2 and Food and Drug Administration (FDA)-approved drug sensitivity test interpretation standards: INH, 0.2 μg/mL; RFP, 1 μg/mL; EMB, 5 μg/mL; OFX, 2 μg/mL; MOX, 0.5 μg/mL; SM, 2 μg/mL; KM, 5 μg/mL; AMK, 4 μg/mL; RFB, 0.5 μg/mL; ETH, 5 μg/mL; PAS, 2 μg/mL; and CS, 0.5 μg/mL (Table S2). If the MIC of a strain was greater than the breakpoint, the strain was considered resistant to the drug. If the MIC was equal or below the breakpoint, the strain was considered sensitive. If the MIC for isoniazid, ethambutol, or streptomycin was higher than 1, 10, or 10 μg/mL, respectively, the strain was considered to have high-level resistance to the relevant drug. For other drugs, if the MIC was at least four times the critical concentration, the strain was considered to have high-level resistance to the drug. Strains with MICs between the breakpoint and four times the critical concentration was considered to have low-level resistance to the drug.

### Statistical analysis.

Excel 2016 was used to summarize the MICs of the strains to each drug and to calculate the number and proportion of drug-resistant strains, the frequency of drug-resistant mutations, and the accuracy of WGS for drug-resistance prediction. The ggplot2 package in *R* version 3.6.0 was used to display the MIC distribution of strains to each drug and the MIC range of strains with each resistance-associated mutation.

### Data availability.

Sequencing data were deposited in the Genome Sequence Archive (https://ngdc.cncb.ac.cn/gsa/) and NCBI under accession numbers CRA005372 and PRJNA819540.
