# Supplementary material for: Whole-Genome Sequencing for Resistance Level Prediction in Multidrug-Resistant Tuberculosis
Source: Microbiol Spectr. 2022 Jun 6;10(3):e02714-21. doi: 10.1128/spectrum.02714-21 (PMC9241708; doi:10.1128/spectrum.02714-21)
Supplement: Supplemental file 1 — Supplemental material. Download spectrum.02714-21-s0001.pdf, PDF file, 1.8 MB [file spectrum.02714-21-s0001.pdf]

Supplementary material

Supplementary Tables

Supplementary Table S1. Drug susceptibility testing results of 13 strains that were excluded because the results of tests determining their minimal inhibitory concentration (MIC) did not agree with results from the proportion method.

Sample	Isoniazid		Rifampicin	
	**MIC (μg/ml)**	**Result of proportion method**	**MIC (μg/ml)**	**Result of proportion method**
TB13067	**0.12**	R	≥16	R
TB14102	**0.12**	R	≥16	R
TB14177	**0.12**	R	≥16	R
TB14229	**0.12**	R	≥16	R
TB14470	**0.06**	R	≥16	R
TB16228	**0.12**	R	≥16	R
TB16246	**0.06**	R	≥16	R
TB18469	**≤0.03**	R	≥16	R
TB14266	≥4	R	**0.5**	R
TB14454	0.12	R	**≤0.12**	R
TB13250	2	R	**0.5**	R
TB14055	0.5	R	**1**	R
TB13051	0.12	R	**1**	R

Note: Values highlighted in boldface correspond to MICs below the critical concentration. The isolates with these values were therefore classified as susceptible.

Supplementary Table S2. The drugs and concentrations tested

Drug	drug concentration (μg/ml)	Critical concentration (μg/ml)
Isoniazid (INH)	0.03, 0.06, 0.12, 0.25, 0.5, 1, 2, 4	0.2
Rifampicin (RIF)	0.12, 0.25, 0.5, 1, 2, 4, 8, 16	1
Ethambutol (EMB)	0.5, 1, 2, 4, 8, 16, 32	5
Streptomycin (SM)	0.25, 0.5, 1, 2, 4, 8, 16, 32	2
Amikacin (AMK)	0.12, 0.25, 0.5, 1, 2, 4, 8, 16	4
Kanamycin (KM)	0.6, 1.25, 2.5, 5, 10, 20, 40	5
Ofloxacin (OFX)	0.25, 0.5, 1, ,2, 4, 8, 16, 32	2
Moxifloxacin (MOX)	0.06, 0.12, 0.25, 0.5, 1, ,2, 4, 8	0.5
Ethionamide (ETH)	0.3, 0.6, 1.25, 2.5, 5, 10, 20, 40	5
Rifabutin (RFB)	0.12, 0.25, 0.5, 1, ,2, 4, 8, 16	0.5
Para-aminosalicylic acid (PAS)	0.5, 1, 2, 4, 8, 16, 32, 64	2
Cycloserine (CS)	2, 4, 8, 16, 32, 64, 128, 256	25

Supplementary Table S3. Statistics of drug-resistant strains based on minimum inhibitory concentration.

Drug	No.	%
**First-line drug resistance**		
Isoniazid	154	100
Rifampin	154	100
Ethambutol	95	61.7
**Other drug resistance**		
Moxifloxacin	52	33.8
Streptomycin	94	61.0
Kanamycin	14	9.1
Amikacin	13	8.4
Rifabutin	105	68.2
Ethionamide	16	10.4

Supplementary Table S4. Frequency of *M. tuberculosis* resistance-conferring mutations.

Drug (No.*)	Gene	Mutation	No. (%)
Isoniazide (152)			
	katG	S315T / S315N	120 (78.9)
		Other	4 (2.6)
	inhA	C-15T	8 (5.3)
		Other	2 (1.3)
	ahpC	*ahpC* promoter mutatiion	2 (1.3)
	katG & inhA	*katG* 315 & inhA promoter mutation	10 (6.6)
		katG 315 & inhA_C-15T& inhA_I21T	1 (0.7)
		Other	4 (2.6)
	katG & ahpC	katG_frameshift & ahpC_C-72T	1 (0.7)
Rifampicin (153)			
	rpoB	S450L / S450W / S450F	94 (61.4)
		H445Y / H445D / H445L / H445N /H445R / H445S	18 (11.8)
		D435V / D435F	6 (3.9)
		Other single mutatiion	5 (3.3)
		S450L & Other	11 (7.2)
		L430P & Other	12 (7.8)
		Other multiple mutations	7 (4.6)
Ethambutol (116)			
	embB		
		M306V / M306I /M306L	67 (57.8)
		G406A / G406S / G406D	17 (14.7)
		Q497R / Q497K	10 (8.6)
		Other single mutatiion	4 (3.4)

		M306I & G406D/D354A/G406A/Q497R	4 (3.4)
	embA	promoter mutation	3 (2.6)
	embA & embB	*embA* promoter & *embB* mutation	11 (9.5)
Moxifloxacin (76)			
	gyrA	D94G / D94A / D94N / D94H / D94Y	41 (53.9)
		A90V	25 (32.9)
		S91P	4 (5.3)
		A90V & D94G	1 (1.3)
	gyrB	A504V	1 (1.3)
	gyrA & gyrB	*gyrA* 94 & *gyrB* mutation	3 (3.9)
		gyrA H70R & *gyrB* E501D	1 (1.3)
Streptomycin (113)			
	rpsL	K43R	73 (64.6)
		K88R	17 (15.0)
	rrs	A514C	10 (8.8)
		Other	3 (2.7)
	gid	gid mutation	4 (3.5)
	rpsL & rrs	*rpsL* & *rrs* mutation	5 (4.4)
	rpsL & gid	*rpsL* K43R & *gid* 115_del_G	1 (0.9)
Amikacin (13)			
	rrs	A1401G	13 (100.0)
kanamycin (20)			
	rrs	A1401G	13 (65.0)
	eis	promoter mutation	7 (35.0)
Rifabutin (152)			
	rpoB	S450L / S450W / S450F	94 (61.8)
		H445Y / H445D / H445L / H445N /H445R / H445S	17 (11.2)
		D435V	6 (3.9)
		Other single mutatiion	5 (3.3)
		S450L & Other	11 (7.2)
		L430P & Other	12 (7.9)
		Other multiple mutations	7 (4.6)
Ethionamide (37)			
	ethA	Framshift	11 (29.7)
		Other	2 (5.4)
	inhA	I21T	1 (2.7)
	fabG1	C-15T	15 (40.5)
		T-8C	2 (5.4)
	fabG1 & ethA	*fabG1* C-15T & *ethA* Frameshift	3 (8.1)
	fabG1 & inhA	*fabG1* C-15T & *inhA* Frameshift	3 (8.1)

*The number of strains with genotypic resistance.

Supplementary Figures

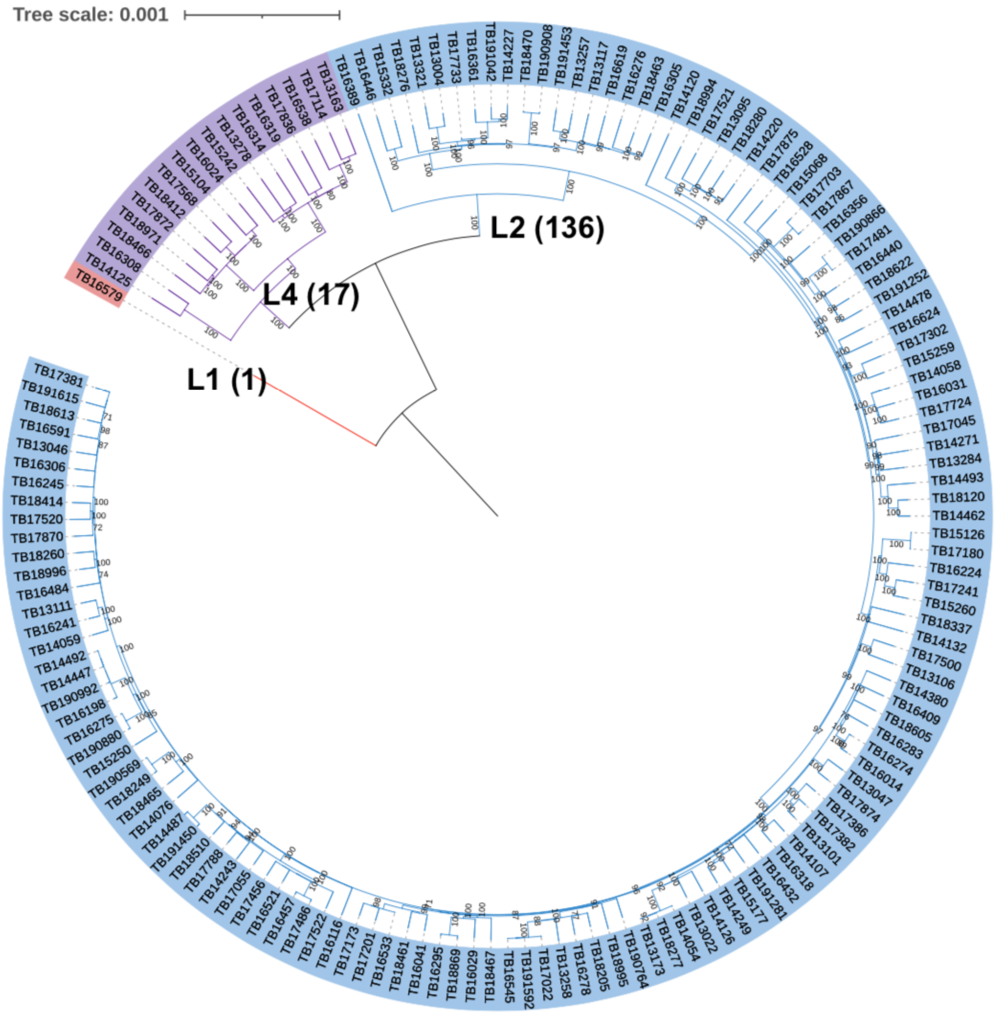

Supplementary Figure S1. The phylogenetic tree of 154 multidrug-resistant tuberculosis strains., Blue indicates Lineage 2 strains, purple indicates Lineage 4 strains, and red indicates Lineage 1 strains.

Isoniazid

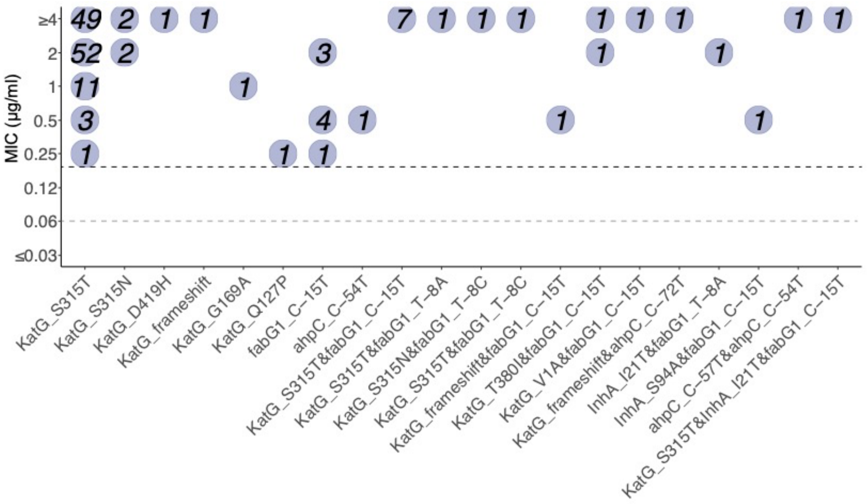

Supplementary Figure S2. Strains with resistance associated mutations and their minimum inhibitory concentrations (MICs) to **isoniazid**. The x axis shows the resistance associated mutations for **isoniazid**, and the y axis, the MICs. The numbers in the circles represent the number of strains that have a particular mutation and MIC. The black dotted line represents the critical concentration of the drug, and the gray line represents the MIC of H37Rv. The promoter of *fabG1* is also that of *inhA*.

Rifampicin

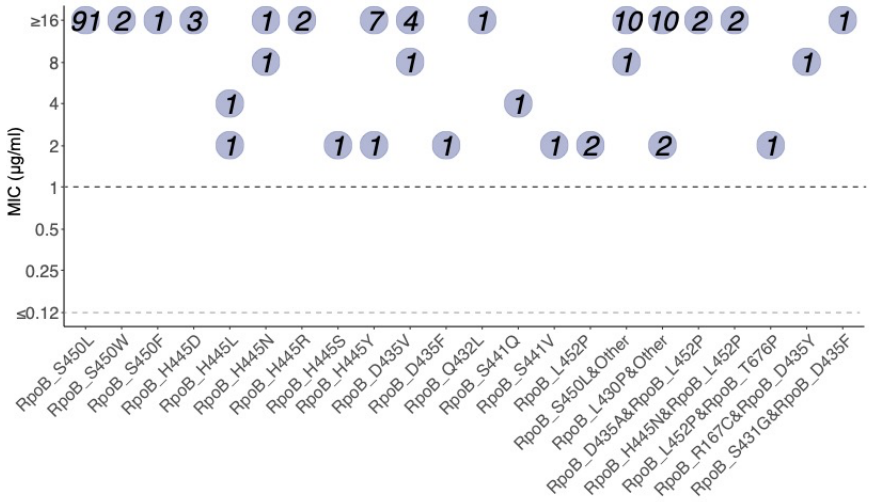

Supplementary Figure S3. Strains with resistance associated mutations and their minimum inhibitory concentrations (MICs) to **rifampicin**. The x axis shows the resistance associated mutations for **rifampicin**, and the y axis, the MICs. The numbers in the circles represent the number of strains that have a particular mutation and MIC. The black dotted line represents the critical concentration of the drug, and the gray line represents the MIC of H37Rv.

Ethambutol

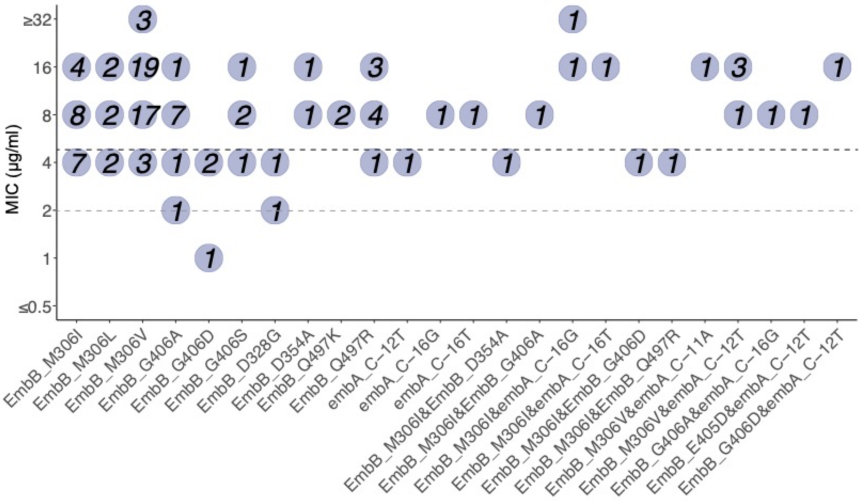

Supplementary Figure S4. Strains with resistance associated mutations and their minimum inhibitory concentrations (MICs) to **ethambutol**. The x axis shows the resistance associated mutations for **ethambutol**, and the y axis, the MICs. The numbers in the circles represent the number of strains that have a particular mutation and MIC. The black dotted line represents the critical concentration of the drug, and the gray line represents the MIC of H37Rv.

Moxifloxacin

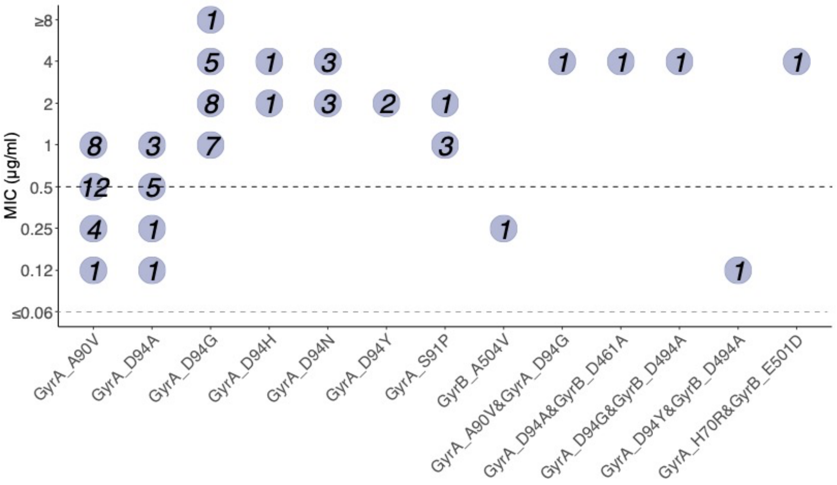

Supplementary Figure S5. Strains with resistance associated mutations and their minimum inhibitory concentrations (MICs) to **moxifloxacin**. The x axis shows the resistance associated mutations for **moxifloxacin**, and the y axis, the MICs. The numbers in the circles represent the number of strains that have a particular mutation and MIC. The black dotted line represents the critical concentration of the drug, and the gray line represents the MIC of H37Rv.

Streptomycin

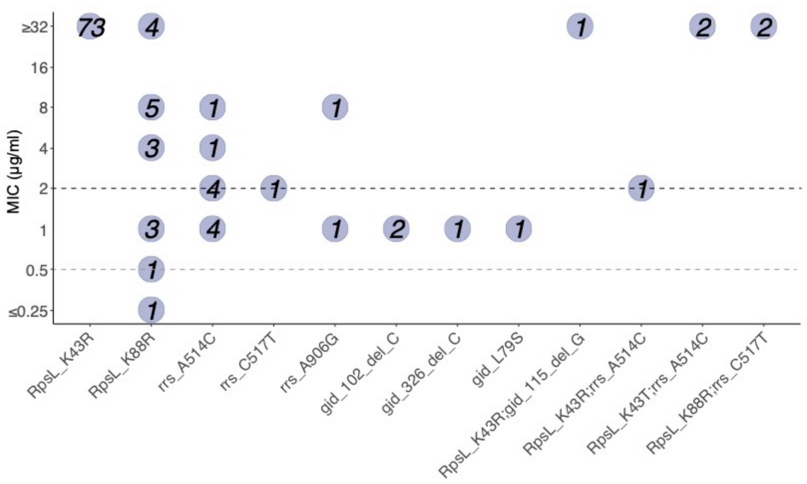

Supplementary Figure S6. Strains with resistance associated mutations and their minimum inhibitory concentrations (MICs) to **streptomycin**. The x axis shows the resistance associated mutations for **streptomycin**, and the y axis, the MICs. The numbers in the circles represent the number of strains that have a particular mutation and MIC. The black dotted line represents the critical concentration of the drug, and the gray line represents the MIC of H37Rv.

Kanamycin

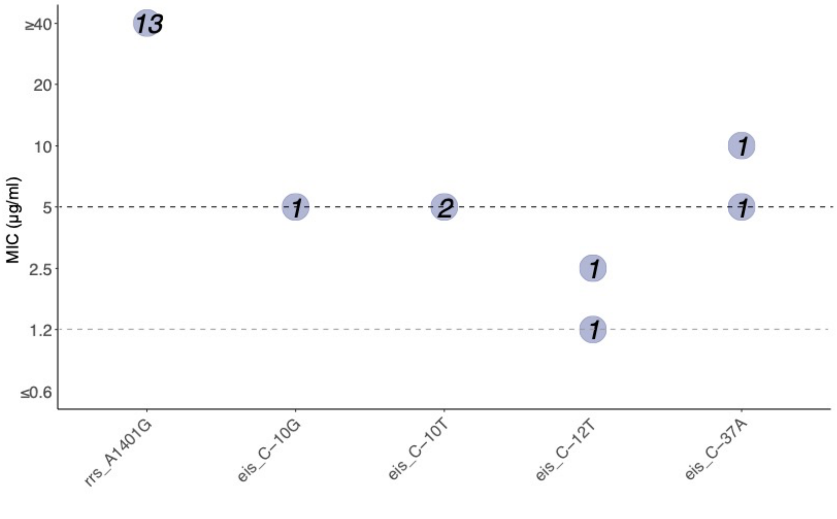

Supplementary Figure S7. Strains with resistance associated mutations and their minimum inhibitory concentrations (MICs) to **kanamycin**. The x axis shows the resistance associated mutations for **kanamycin**, and the y axis, the MICs. The numbers in the circles represent the number of strains that have a particular mutation and MIC. The black dotted line represents the critical concentration of the drug, and the gray line represents the MIC of H37Rv.

Ethionamide

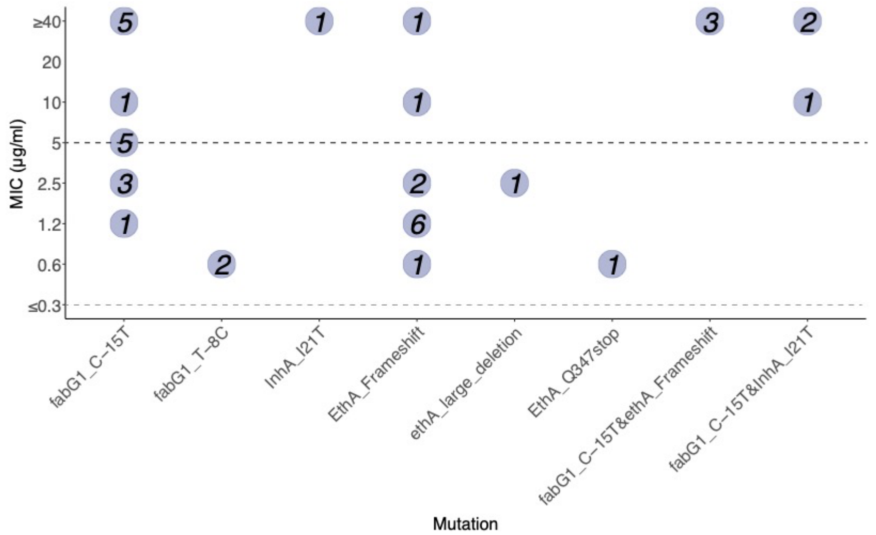

Supplementary Figure S8. Strains with resistance associated mutations and their minimum inhibitory concentrations (MICs) to **ethionamide**. The x axis shows the resistance associated mutations for **ethionamide**, and the y axis, the MICs. The numbers in the circles represent the number of strains that have a particular mutation and MIC. The black dotted line represents the critical concentration of the drug, and the gray line represents the MIC of H37Rv.

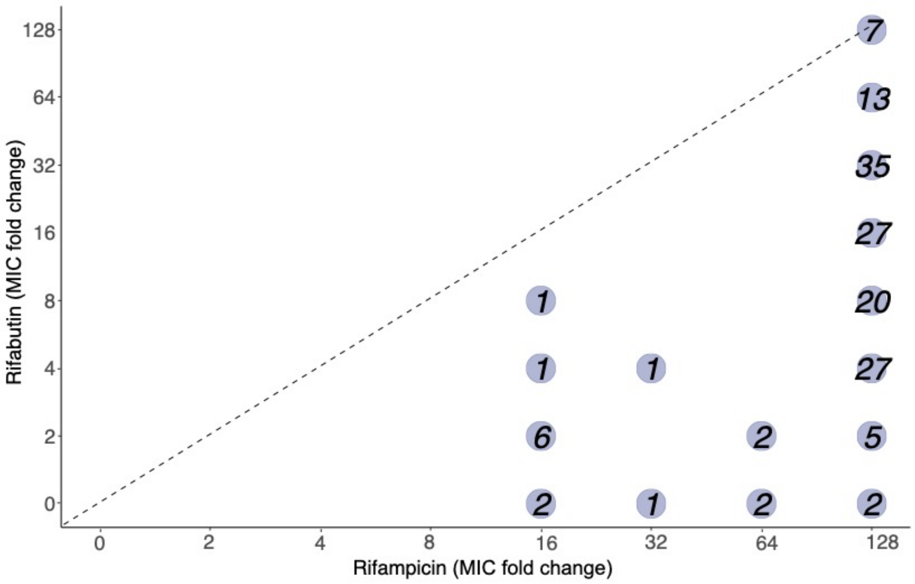

Supplementary Figure S9. Fold change in minimal inhibitory concentrations (MICs) to rifampicin and rifabutin for strains with RIF resistance mutations compared to H37Rv.

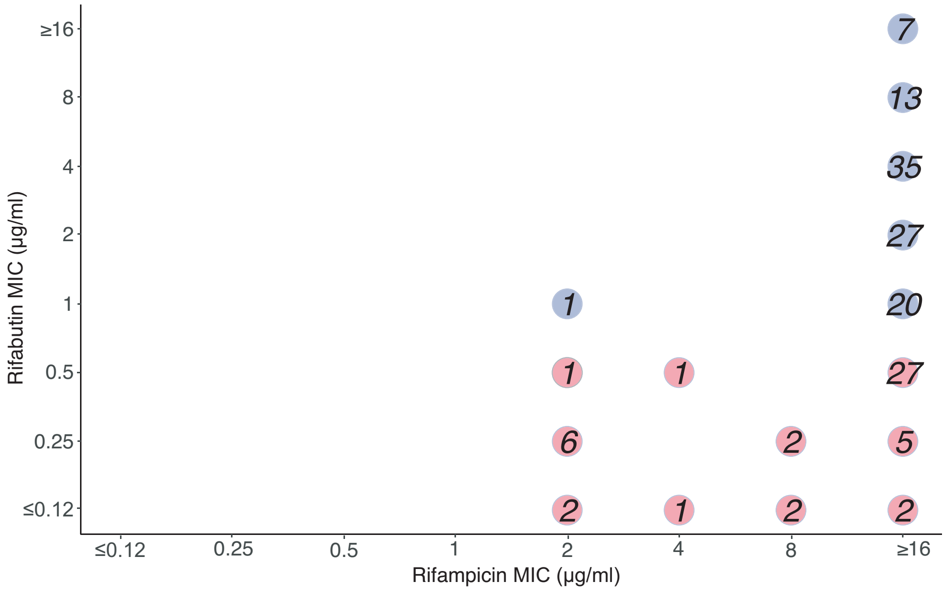

Supplementary Figure S10. The minimal inhibitory concentrations (MICs) to rifampicin and rifabutin for strains with RIF resistance mutations. The x axis and y axis show the MICs for rifampicin and rifabutin. The numbers in the circles represent the number of strains with certain MICs to the two drugs. The pink background indicates the rifampicin-resistant/rifabutin-sensitive ones.

Rifabutin

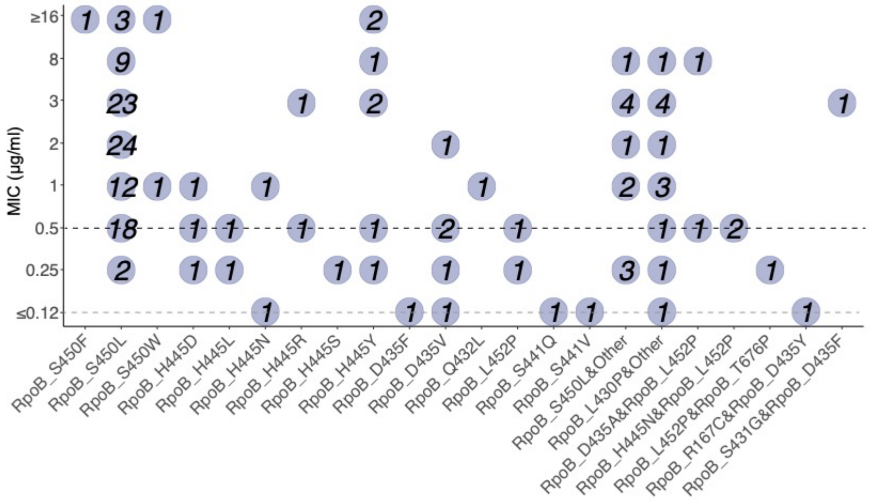

Supplementary Figure S11. Strains with resistance associated mutations and their minimum inhibitory concentrations (MICs) to **rifabutin**. The x axis shows the resistance associated mutations for **rifabutin**, and the y axis, the MICs. The numbers in the circles represent the number of strains that have a particular mutation and MIC. The black dotted line represents the critical concentration of the drug, and the gray line represents the MIC of H37Rv.
